# PFGE, Lior serotype, and antimicrobial resistance patterns among *Campylobacter jejuni *isolated from travelers and US military personnel with acute diarrhea in Thailand, 1998-2003

**DOI:** 10.1186/1757-4749-2-15

**Published:** 2010-11-10

**Authors:** Oralak Serichantalergs, Piyarat Pootong, Anders Dalsgaard, Ladaporn Bodhidatta, Patricia Guerry, David R Tribble, Sinn Anuras, Carl J Mason

**Affiliations:** 1 Department of Enteric Diseases, Armed Forces Research Institute of Medical Sciences, 315/6 Rajvithi Road, Phayathai, Bangkok 10400, Thailand; 2 Department of Veterinary Pathobiology, Faculty of Life Sciences, Copenhagen University, Stigboejlen 4, DK-1870 Frederiksberg C, Denmark; 3 Naval Medical Research Center, 503 Robert Grant Ave, Silver Spring, MD 20910, USA; 4 Uniformed Services University of the Health Sciences, 4301 Jones Bridge Road, Bethesda, MD 20814-5119, USA; 5 Bumrungrad Hospital, 33 Sukhumvit Soi 3, Bangkok 10110, Thailand

## Abstract

**Background:**

*Campylobacter jejuni *is a major cause of gastroenteritis worldwide. In Thailand, several strains of *C. jejuni *have been isolated and identified as major diarrheal pathogens among adult travelers. To study the epidemiology of *C. jejuni *in adult travelers and U.S. military personnel with acute diarrhea in Thailand from 1998-2003, strains of *C. jejuni *were isolated and phenotypically identified, serotyped, tested for antimicrobial susceptibility, and characterized using pulsed-field gel electrophoresis (PFGE).

**Results:**

A total of 312 *C. jejuni *isolates were obtained from travelers (n = 46) and U.S. military personnel (n = 266) in Thailand who were experiencing acute diarrhea. Nalidixic acid and ciprofloxacin resistance was observed in 94.9% and 93.0% of the isolates, respectively. From 2001-2003, resistance to tetracycline (81.9%), trimethoprim-sulfamethoxazole (57.9%), ampicillin (28.9%), kanamycin (5.9%), sulfisoxazole (3.9%), neomycin (2.0%), and streptomycin (0.7%) was observed. Combined PFGE analysis showed considerable genetic diversity among the *C. jejuni *isolates; however, four PFGE clusters included isolates from the major Lior serotypes (HL: 36, HL: 11, HL: 5, and HL: 28). The PFGE analysis linked individual *C. jejuni *clones that were obtained at U.S. military exercises with specific antimicrobial resistance patterns.

**Conclusions:**

In summary, most human *C. jejuni *isolates from Thailand were multi-resistant to quinolones and tetracycline. PFGE detected spatial and temporal *C. jejuni *clonality responsible for the common sources of *Campylobacter *gastroenteritis.

## Background

*Campylobacter jejuni *is a major cause of gastroenteritis worldwide, especially in children, travelers, and military personnel deployed to developing countries [1-4]. In recent years, a high prevalence of infection and an increased resistance to the antimicrobials used to treat diarrhea have been documented [5,6]. *C. jejuni *and *C. coli *can be phenotypically characterized by growth characteristics, biochemical reactions, and hippurate hydrolysis [7]. Serotyping techniques for *C. jejuni *and *C. coli *have been developed [8-10]. Molecular techniques such as RFLP, RAPD, PFGE, AFLP, and MLST have also been applied to *C. jejuni *isolate characterization [11].

PFGE is a well-known technique standardized by the Centers for Disease Control and Prevention (CDC) for subtyping *Salmonella *spp., *Shigella *spp., and *Vibrio *spp., in addition to *C. jejuni *[12,13]. Unlike other enteric bacteria, *Campylobacter *is a genetically diverse organism that undergoes intra- and inter-genomic exchange. However, PFGE is considered to be the most discriminatory method of characterizing *C. jejuni *and *C. coli *and identifying specific *Campylobacter *spp. in outbreak studies [14-16]. Furthermore, the combination of PFGE and other typing techniques can identify common sources of *Campylobacter *and other bacterial infections [17-19].

In Thailand, *C. jejuni *was isolated and identified as a major diarrheal pathogen among children and adult travelers, including U.S. military personnel [20-23]. A high prevalence of infection with fluoroquinolone-resistant *C. jejuni *was previously reported in both Thai children and U.S. military personnel [3,24]. However, epidemiological data on *Campylobacter *infection among travelers and expatriates as well as their susceptibility to other antimicrobials have not been described in Thailand in several years. This prompted us to investigate and characterize the *C. jejuni *isolates responsible for gastroenteritis in adult travelers by combining antimicrobial resistance data, serotype classification, and PFGE.

## Results and Discussion

### Frequency and distribution of *C. jejuni *serotypes

From a total of 312 *C. jejuni *isolates obtained in this study, 266 isolates were from U.S. soldiers and 46 were from foreign travelers seen at Bumrungrad Hospital, Bangkok. The prevalence of *C. jejuni *from diarrheal cases was detected as 11.0% (46/417) in the Bumrungrad Hospital study and 30.4% (266/875) in samples from U.S. military personnel in the Cobra Gold exercises of 1998-2003 (Table 1). Overall, a total of 16 Lior serotypes were detected; the three most common serotypes were HL: 36, HL: 11, and HL: 5; these serotypes accounted for 25.0% (78/312), 13.8% (43/312), and 7.4% (23/312) of all of the isolates, respectively. Untypable strains composed 17.6% (55/312) of the isolates. The distribution of the serotypes and the incidence of *C. jejuni *isolates among foreign travelers and U.S. military personnel are shown in Table 2. The two most common serotypes by study location and year are shown in Table 1.

**Table 1:** Numbers of *C. jejuni *isolates and two most common Lior serotypes by study location/year

Year	Study location	Enrolled diarrhea cases	Number of *C. jejuni *isolates	Two most common Lior serotypes
		(Total = 1292)	(Total = 312)	(Number of isolates)
1998	Kanjanaburi	166	10	HL: 11 (3), HL: 36 (2)
	Cholburi		12	HL: 36 (3), HL: 11 (2)
1999	Korat	198	84	HL: 36 (33), HL: 19 (15)
2000	Nakornsrithammarat	256	68	HL: 11 (23), HL: 36 (19)
2001	Phitsanulok	153	54	HL: 5 (11), HL: 36 (8)
2001-2002	Bangkok	417	46	untypable (35), HL:11 (5)
2002	Sakaew	54	15	HL: 36 (7), untypable (4)
2003	Pranburi	48	23	HL: 4 (7), HL: 36 (3)

**Table 2:** Lior serotype distribution of *C. jejuni *isolates among foreign travelers and U.S. military personnel (1998-2003)

Lior serotype	Serotypes in foreign travelers % (*n*)	Serotypes in U.S. military personnel % (*n*)	% Total (*n*)
	(Total = 46)	(Total = 266)	(Total = 312)
HL: 36	6.5 (3)	28.2 (75)	25.0 (78)
HL: untypable	54.3 (25)	11.7 (30)	17.6 (55)
HL: 11	6.5 (3)	14.7 (40)	13.8 (43)
HL: 5	4.3 (2)	7.9 (21)	7.4 (23)
HL: 1	0.0	7.5 (19)	6.1 (19)
HL: 4	4.3 (2)	5.6 (15)	5.4 (17)
HL: 19	0.0	6.0 (17)	5.4 (17)
HL: 7	2.2 (1)	4.9 (13)	4.5 (14)
HL: 114	4.3 (2)	4.1 (10)	3.8 (12)
HL: 28	4.3 (2)	3.8 (10)	3.8 (12)
HL: 102	2.2 (1)	1.9 (5)	1.9 (6)
HL: 2	0.0	1.9 (5)	1.6 (5)
HL: 9	0.0	1.5 (4)	1.3 (4)
HL: 17	4.3 (2)	0.4 (1)	1.0 (3)
HL: 41	2.2 (1)	0.0	0.3 (1)
HL: 53	0.0	0.4 (1)	0.3 (1)
HL: 6	2.2 (1)	0.0	0.3 (1)
HL: 86	2.2 (1)	0.0	0.3 (1)

The incidence of the serotypes described herein were included from the first to tenth ranks among the global isolates [25] but were slightly different from those reported previously in Thai children [24]. For example, serotype HL: 5 was detected infrequently among Thai children [24]. In this study, serotype HL: 5 was more common, and serotypes HL: 9 and HL: 2 were less common. The majority of *C. jejuni *isolates from serotype HL: 5 (11/23) were isolated in 2001, and most *C. jejuni *HL: 19 isolates (15/17) were obtained in 1999. The untypable isolates comprised 54.3% (25/46) of the isolates from foreign travelers in 2001-2002 compared to 11.3% (30/266) of the isolates from U.S. military personnel in this study. The adult travelers from Bumrungrad Hospital spanned several nationalities, including Japanese, European, American, and Australian. The high percentage of untypable isolates might reflect the diverse foods consumed by these travelers, while the low percentage of untypable isolates in the military personnel might reflect the limited diet consumed by U.S. military personnel on deployment.

### PFGE analysis of *C. jejuni *isolates

**Figure 1:**
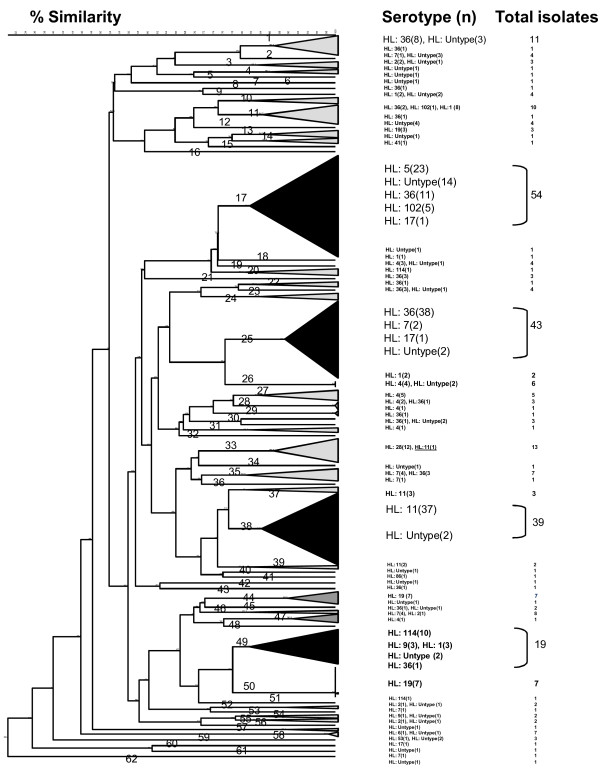
**Dendrogram and Lior serotype of 312 *C. jejuni *isolates from travelers' diarrhea in Thailand (1998-2003)**. PFGE cluster analysis of a *Sma*I/*Kpn*I restriction enzyme digest of genomic DNA from 312 *C. jejuni *isolates analyzed at an 80% similarity cut off, resulting in 62 genotypes distributed between Lior serotypes.

At an 80% similarity level, a dendrogram combining data for all 312 *C. jejuni *isolates from Thailand was clustered into 62 genotypes (Figure 1). Four major genotypes composed 49.7% (155/312) of the *C. jejuni *isolates in this study, and 30 of the 62 genotypes included only a single *C. jejuni *isolate. Of the 55 isolates that were untypable by Lior serotyping, 42 could be grouped into a genotype with other known serotypes.

### Antimicrobial susceptibility tests

In 1998-2003, 94.9% (296/312) and 93.0% (289/311) of the isolates were resistant to quinolones (NAL and CIP), but 99.0% (306/309) were susceptible to macrolides (ERY and AZM). The high prevalence of quinolone (NAL and CIP) resistance and macrolide (ERY and AZM) sensitivity is consistent with previously reported results from Thailand [21,24,26]. For the 138 *C. jejuni *isolates from 2001-2003, the percentages of resistant isolates detected were as follows (Table 3): CF, 100% (138/138); TE, 81.9% (113/138); SXT, 58.0% (80/138); AMP, 30.4% (42/138); KM, 6.5% (9/138); SU, 3.6% (5/138); NM, 2.2% (3/138); and SM, 0.7% (1/138). None of these 138 isolates were resistant to GM, CM or CL. Notably, tetracycline resistance was also detected in over 80% of isolates that were similar to *C. jejuni *isolates from Taiwan (95%) [27], Korea (87%) [6,28], Canada, and the U.S. (50%) [29,30].

**Table 3:** Antimicrobial resistance among 138 human *C. jejuni *isolates from Thailand (2001-2003)

Antimicrobial	Percentage of resistant isolates (number of isolates)				
	
	Phitsanulok 2001 (*n *= 54)	Bangkok 2001-2002 (*n *= 46)	Sakaew 2002 (*n *= 15)	Pranburi 2003 (*n *= 23)	p-value *
Nalidixic acid	96.2% (52)	93.5% (43)	86.6% (13)	100.0% (23)	*NS*
Ciprofloxacin	96.2% (52)	87.0% (40)	86.6% (13)	91.3% (21)	*NS*
Erythromycin	0.0% (0)	6.5% (3)	0.0% (0)	0.0% (0)	*NS*
Azithromycin	0.0% (0)	6.5% (3)	0.0% (0)	0.0% (0)	*NS*
Tetracycline	90.7% (49)	76.0% (35)	86.6% (13)	69.5% (16)	*NS*
Trimethoprim-sulfamethoxazole	90.7% (49)	58.7% (27)	26.7% (4)	0.0% (0)	<0.001
Ampicillin	31.5% (17)	30.4% (14)	60.0% (9)	8.7% (2)	<0.05
Kanamycin	0.0% (0)	2.2% (1)	20.0% (3)	21.8% (5)	<0.001
Sulfisoxazole	0.0% (0)	10.9% (5)	0.0% (0)	0.0% (0)	NA
Neomycin	0.0% (0)	2.2% (1)	1.7% (1)	1.7% (1)	NA
Streptomycin	0.0% (0)	0.0% (0)	1.7% (1)	0.0% (0)	NA
Gentamicin	0.0% (0)	0.0% (0)	0.0% (0)	0.0% (0)	NA
Colistin	0.0% (0)	0.0% (0)	0.0% (0)	0.0% (0)	NA
Chloramphenicol	0.0% (0)	0.0% (0)	0.0% (0)	0.0% (0)	NA
Cephalothin	100.0% (52)	100.0% (60)	100.0% (15)	100.0% (23)	NA

* Pearson chi-square Monte Carlo (two-tailed); *NS*, non-significant; NA, not applicable

The two most common resistance patterns observed in these 138 isolates were multiple resistance to four antimicrobials (NAL, CIP, CF, and TE), observed in 79.0% (109/138) of the isolates, and multiple resistance to five antimicrobials (NAL, CIP, CF, TE, and SXT), detected in 47.8% (66/138) of the isolates. Another resistance pattern (NAL, CIP, CF, TE, SXT, and AMP) was found in 15.9% (22/138) of the isolates, and a fourth pattern of resistance (NAL, CIP, CF, TE, and KM) was detected in 5.8% (8/138) of the isolates. The first common pattern was similar to a previous report of antimicrobial resistance to NAL, CIP, and TE in 53% of clinical *C. jejuni *isolates in Thailand [31]. These results confirm widespread quinolone and tetracycline resistance among *C. jejuni *isolates from traveler's diarrhea in Thailand.

Antimicrobial resistance by study location and the results of the chi-square test are shown in Table 3. Interestingly, KM-resistant isolates were detected in 6.5% (9/138) of the isolates, but there was a significant difference (from 2.2% to 21.8%) in the frequency of resistance in isolates from different locations (p < 0.001). The percentage of AMP-resistant isolates also varied by location (p < 0.05). The resistance of isolates to SXT varied greatly from 90.7% at Phitsanulok to 58.7% at Bumrungrad Hospital, 26.7% at Sakaew, and to undetectable levels in isolates from Pranburi (p < 0.001). The finding of differences in AMP, KM, and SXT resistance among *C. jejuni *isolates from selected sites should not be considered as indicative of significant changes over time in Thailand.

In this study, the antimicrobial susceptibility tests were performed by disk diffusion assay. Other methods, including agar dilution and broth micro dilution methods, and epsilometer test (E-tests) have been used by different laboratories to measure antimicrobial susceptibilities for *Campylobacter *spp. [32-35]. However, good agreement of antimicrobial susceptibility test between disk diffusion and agar dilution tests has been observed in several classes of antimicrobials especially quinolone/fluoroquinolones and aminoglycosides suggesting that disk diffusion test could be used as qualitative assay but not quantitative assay for antimicrobial susceptibility among *Campylobacter *spp. [36]. Although another study suggested that interpretation of erythromycin by disk diffusion assay was unreliable and should be confirmed by MIC-based methods [37], but our previous data showed high correlation of antimicrobial susceptibility by disk diffusion and agar dilution tests in erythromycin and azithromycin-resistant *C. jejuni *and *C. coli *isolates [38].

### PFGE and antimicrobial resistance by study location

**Figure 2:**
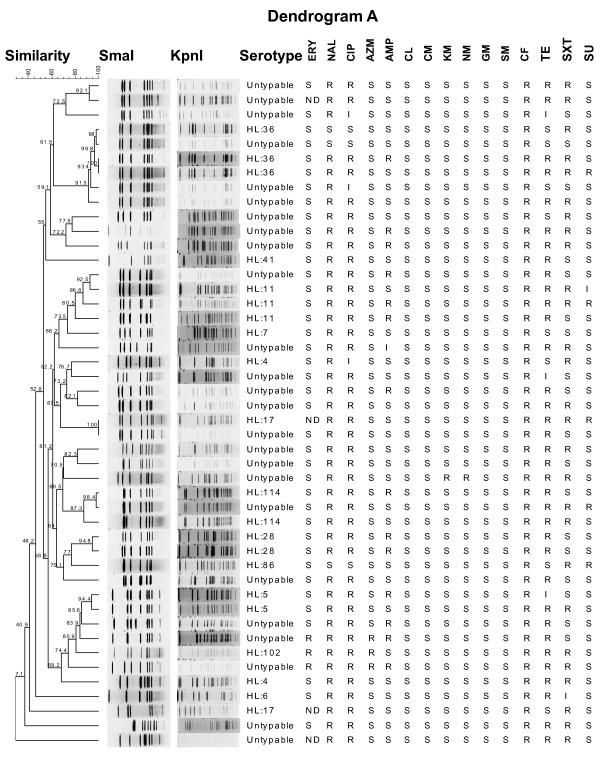
**Dendrogram of *C. jejuni *isolates with Lior serotypes and antimicrobial resistance from Bumrungrad Hospital (2001-2002)**. Dendrogram A: PFGE cluster analysis of a *Sma*I/*Kpn*I restriction enzyme digest of genomic DNA from 46 *C. jejuni *isolates with marked clonal heterogeneity, unrelated antimicrobial resistance patterns, and 25 untypable isolates from this study site. R, resistance; I, intermediate susceptibility; S, susceptible; ND, not tested.

**Figure 3:**
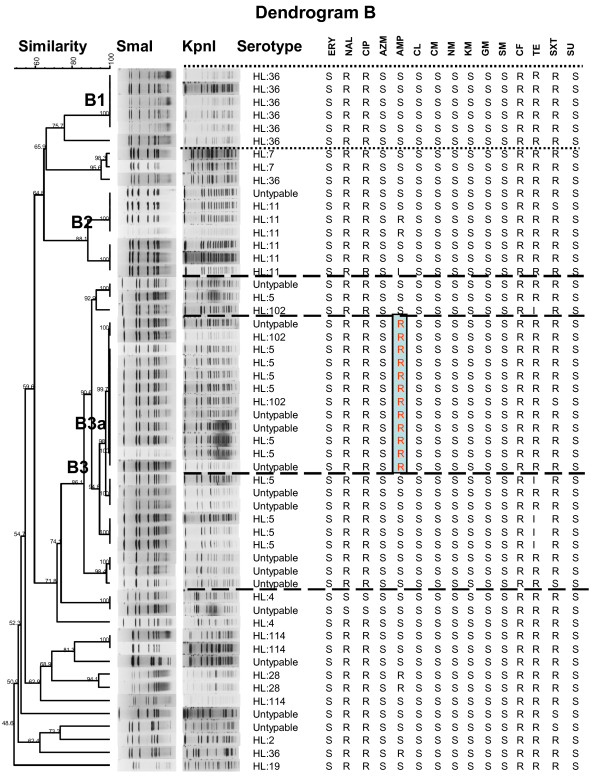
**Dendrogram of 54 *C. jejuni *isolates with Lior serotypes and antimicrobial resistance from Phitsanulok 2001**. Dendrogram B: PFGE cluster analysis of a *Sma*I/*Kpn*I restriction enzyme digest of genomic DNA from 54 *C. jejuni *isolates from the Cobra Gold exercises (Phitsanulok, 2001) with evidence of clonality due to similarity in the PFGE patterns, Lior serotype(s) and related specific antimicrobial susceptibility patterns (genotypes B1 and B3a). R, resistance; I, intermediate susceptibility; S, susceptible.

**Figure 4:**
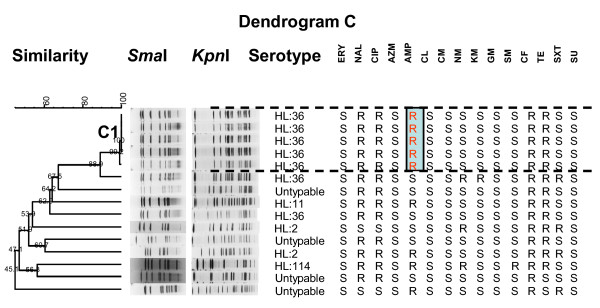
**Dendrogram of 15 *C. jejuni *isolates with Lior serotypes and antimicrobial resistance from Sakaew 2002**. Dendrogram C: PFGE cluster analysis of a *Sma*I/*Kpn*I restriction enzyme digest of genomic DNA of 15 *C. jejuni *isolates from the Cobra Gold exercises (Sakaew, 2002) with evidence of clonality due to similarity in the PFGE patterns (>99.2%), identical Lior serotype (HL:36) and a specific antimicrobial susceptibility pattern (genotype C1 with ampicillin resistance). R, resistance; S, susceptible.

**Figure 5:**
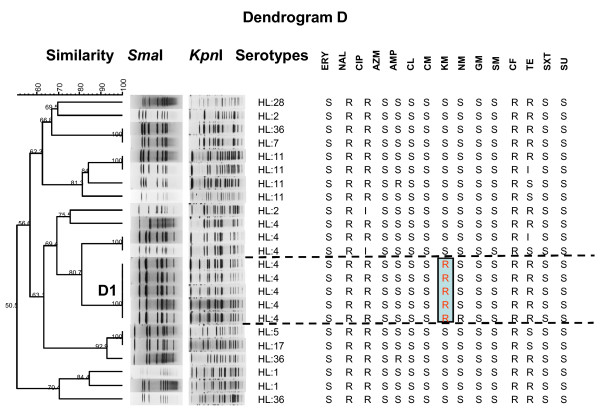
**Dendrogram of 23 *C. jejuni *isolates with Lior serotypes and antimicrobial resistance from Pranburi 2003**. Dendrogram D: PFGE cluster analysis of a *Sma*I/*Kpn*I restriction enzyme digest of genomic DNA of 23 *C. jejuni *isolates from the Cobra Gold exercises (Pranburi, 2003) with evidence of clonality due to similarity in the PFGE patterns (100%), identical Lior serotype (HL: 4) and specific antimicrobial susceptibility patterns (genotype D1 with additional kanamycin resistance). R, resistance; I, intermediate susceptibility; S, susceptible.

Dendrograms A thru D in Figs. 2, 3, 4, and 5 were generated for four study locations from 2001-2003. Dendrogram A includes 46 isolates obtained from Bumrungrad Hospital, Bangkok, over the two-year period from 2001-2002 (Figure 2). Although the common resistance pattern (NAL, CIP, CF, and TE) was observed in 76.1% (35/46) of the isolates, there was substantial heterogeneity in the PFGE patterns (Figure 2). The PFGE analysis suggests diverse genotypes. No specific multi-resistant antimicrobial patterns were noted in the remainder of these isolates. The dendrogram and the diversity of serotypes and antimicrobial susceptibility patterns suggest that these isolates are heterogeneous and clonally diverse.

Dendrogram B includes the 54 isolates collected from U.S. soldiers in Phitsanulok in 2001 (Figure 3). As above, 87% (47/54) of these isolates were generally multi-resistant to NAL, CIP, CF, and TE; in addition, 87% (47/54) were resistant to NAL, CIP, CF, and SXT. The genotype B3 included 24 isolates exhibiting 86.1% similarity that belonged to three serotypes: HL: 5 (11), HL: 102 (3), and HL: untypable (10). A subset of 12 isolates (B3a) within the genotype B3 had 99.7% similarity and resistance to AMP, suggesting clonality (Figure 3). Similarly, genotype B1 consisted of five isolates of serotype HL: 36 with 100% similarity and an identical antimicrobial resistance pattern (NAL, CIP, CF, TE, and SXT). *C. jejuni *isolates in genotypes B1 and B3a were isolated over a 5-day period and 8-day period, respectively.

Dendrogram C (Figure 4) shows 15 isolates collected from U.S. soldiers in Sakaew over a 1-month period in 2002. Genotype C1 includes 5 isolates of serotype HL: 36 with 99.2% similarity and an identical antimicrobial resistance pattern (NAL, CIP, CF, AMP, and TE), which also suggests clonality. The other 10 isolates had <67.5% similarity and included five serotypes and seven antimicrobial resistance patterns, suggesting more diverse sources (Figure 4). All *C. jejuni *isolates in genotype C1 were isolated over a period of 6 days.

Dendrogram D (Figure 5) shows the 23 isolates collected from U.S. soldiers in Pranburi during a 1-month exercise in 2003 and genotype D1 includes a unique cluster of five serotype HL: 4 isolates with an unusual multi-resistant antimicrobial pattern (NAL, CIP, CF, KM, and TE), suggesting clonality for these isolates. Similar to dendrograms B and C, genotype D1 included *C. jejuni *isolates collected during a 10-day period.

## Conclusions

In summary, our study demonstrates the usefulness of PFGE in local epidemiological studies or in the study of small outbreaks occurring over a short time interval rather than in the long-term epidemiological studies that have been studied by others [26]. This study confirmed the existence of common *C. jejuni *clones that are associated with specific serotypes and multiple antimicrobial resistance patterns in the Cobra Gold military exercises but not in the traveler's diarrhea study at Bumrungrad Hospital. A possible explanation for these findings is that the Cobra Gold military exercises took place at particular locations with short durations (1 month). Diarrhea cases among soldiers might also be expected to be caused by common exposures. Our finding of an association between PFGE and serotype with the antimicrobial resistance patterns in these exercises differed from other studies in which the correlation between PFGE and multi-antimicrobial resistance was low [28,39,40]. In the Bumrungrad Hospital study, where diarrhea cases occurred in diverse populations over a 2-year period, a similar low correlation was observed between serotype and antimicrobial resistance. Our data suggest that these patients became infected with unrelated *C. jejuni *isolates. Comprehensive monitoring of human *C. jejuni *isolates, including animal and environmental sources, should be expanded in Thailand to monitor antimicrobial resistance and to better document potential sources of infection.

## Methods

### Sources of isolates

Under approved human use protocols, stool specimens were obtained from patients with diarrhea and from asymptomatic controls in an acute diarrhea study among foreign travelers from highly developed countries at Bumrungrad Hospital in Bangkok, Thailand during 2001-2002. Stool specimens were collected from U.S. soldiers with acute diarrhea and from asymptomatic controls; the soldiers were deployed for the Cobra Gold exercises lasting one for four weeks at different sites in Thailand during 1998-2003. Only *C. jejuni *isolates from acute diarrhea cases were included in this study. Table 1 describes the number of *C. jejuni *isolates from cases in each study location.

### Isolation and identification

All stool specimens were cultured for *Campylobacter *spp. using a modified filtration method described previously [41]. Suspected colonies, growing on Brucella Agar (Difco, Detroit, MI, USA) with 5% sheep blood (BAP), were identified as *Campylobacter *spp. by colony characteristics, Gram staining, oxidase tests, and catalase tests, followed by phenotypic tests including hippurate hydrolysis, nitrate reduction, H_2_S TSI, oxygen tolerance, and microaerobic growth at 25°C, 37°C, and 42°C. *C. jejuni *isolates were differentiated from *C. coli *by the hippurate hydrolysis test. All *C. jejuni *isolates were kept in glycerol medium at -70°C for further analysis.

### Serotyping

Lior serotyping was performed by an agglutination assay with specific antiserum obtained from the National Laboratory for Enteric Pathogens (NLEP) in Winnipeg, Manitoba, Canada. These antisera were routinely used to serotype *C. jejuni *and *C. coli *isolates at AFRIMS. The antisera detect heat-labile antigens [9] and identify 33 common HL serotypes.

### Antimicrobial susceptibility testing

*C. jejuni *isolates were tested for susceptibility to antimicrobial drugs using a disk diffusion assay as described previously [42], with modifications. BAP subcultures of patient isolates at 18- to 48-h were suspended in Mueller Hinton broth (BD Diagnostic Systems, Sparks, MD, USA.) to obtain a turbidity equivalent to a 1.0 McFarland standard, and suspensions were inoculated onto Mueller Hinton II agar supplemented with 5% sheep blood. At the time of each study, all *C. jejuni *isolates were tested for susceptibility to the following antimicrobials (BD Diagnostic Systems): NAL (30 μg), CIP (5 μg), ERY (15 μg), and AZM (15 μg). The 138 *C. jejuni *isolates in 2001-2003 were further tested for susceptibility to 11 additional antimicrobials by disk diffusion assay. These antimicrobial disks included AMP (10 μg), CM (30 μg), KM (30 μg), GM (10 μg), SM (10 μg), TE (30 μg), SXT (1.25/23.75 μg), SU (250 μg), CL (10 μg), NM (30 μg), and CF (30 μg). Disks were placed on the surfaces of inoculated Mueller Hinton II agar plates. Inoculated plates were incubated at 37°C for 24 h in a microaerobic environment. The plates were re-incubated up to 48 h if insufficient growth of *C. jejuni *isolates on the Muller Hinton II agar plates was obtained at 24 h. Because no standardized interpretive criteria exist for *Campylobacter *spp., the inhibition zone diameters were measured and interpreted following the disk manufacturer's instructions and compared against the Clinical and Laboratory Standards Institute (formerly NCCLS) standard guidelines for aerobic gram-negative bacilli to interpret the results as susceptible, intermediate, or resistant [43]. *Escherichia coli *ATCC 25922 and *Staphylococcus aureus *ATCC 25923 were used as standard organisms for all disk diffusion assays.

### Statistical analysis

Pearson's chi-square tests of independence for the antimicrobial susceptibility data (NAL, CIP, ERY, AZM, TE, SXT, AMP, and KM) between locations were performed using the Monte Carlo-exact (2-sided) method in SPSS version 12.0 (SPSS Inc., Chicago, IL, USA). A p-value < 0.05 was considered significant.

### PFGE

PFGE was performed according to the One-Day (24-28 h) Standardized Laboratory Protocol for Molecular Subtyping by the CDC [12] with the minor modifications described below. The cell density of each isolate was adjusted to an O.D. of 0.6 using a spectrophotometer (Spectramax 190; Molecular Devices, Sunnyvale, CA, USA) that was different from the spectrophotometers suggested by the CDC. The PFGE patterns were analyzed to generate dendrograms of the combined *Sma*I and *Kpn*I similarities using BioNumerics version 5.0 (Applied Maths, Sint-Martens-Latem, Belgium) by UPGMA type and Dice coefficient with 1.5% optimization and tolerance. Dendrograms were made for the composite data of all of the *C. jejuni *isolates and for the different locations.

## Abbreviations

The following abbreviations were used: RFLP: restriction fragment length polymorphism; RAPD: random amplification of polymorphic DNA; PFGE: pulsed-field gel electrophoresis; AFLP: amplified fragment length polymorphism; MLST: multilocus sequence typing; MIC: minimal inhibitory concentration; HL: heat-labile; NAL: nalidixic acid; CIP: ciprofloxacin; ERY: erythromycin; AZM: azithromycin; AMP: ampicillin; CM: chloramphenicol; KM: kanamycin; GM: gentamicin; SM: streptomycin; TE: tetracycline; SXT: trimethoprim-sulfamethoxazole; SU: sulfisoxazole; CL: colistin; NM: neomycin; CF: cephalothin.

## Competing interests

The authors declare that they have no competing interests.

## Authors' contributions

OS designed and carried out the study project (including the data analysis and preparation of the draft manuscript), PP performed the PFGE of the *C. jejuni *isolates, AD provided ideas and comments on the draft manuscript, LB analyzed the epidemiological data for the *C. jejuni *isolates, PG provided expertise on the molecular biology of *C. jejuni*, DT directed the patient recruitment in the US military exercise, SA supported the study project etiology of acute diarrhea at Bumrungrad Hospital, and CJM conceived the idea for the study (and performed statistical analysis and worked on the final manuscript). All authors read and approved the final manuscript.
